# 3-(4-Chloro­phen­yl)-1-[(*E*)-1-(4-chloro­phen­yl)-2-(4-methyl­phenyl­sulfan­yl)ethen­yl]-4-(4-methyl­phenyl­sulfan­yl)-1*H*-pyrazole

**DOI:** 10.1107/S1600536809043293

**Published:** 2009-10-28

**Authors:** P. Ramesh, Ramaiyan Manikannan, S. Muthusubramanian, S. S. Sundaresan, M. N. Ponnuswamy

**Affiliations:** a Centre of Advanced Study in Crystallography and Biophysics, University of Madras, Guindy Campus, Chennai 600 025, India; b Department of Organic Chemistry, School of Chemistry, Madurai Kamaraj University, Madurai 625 021, India

## Abstract

In the title compound, C_31_H_24_Cl_2_N_2_S_2_, the pyrazole ring adopts planar conformation with a maximum deviation of 0.002 (2) Å. The chloro­phenyl rings are twisted out of the plane of the pyrazole ring by 75.1 (1) and 39.5 (1)°. The crystal packing is controlled by weak intermolecular C—H⋯π interactions.

## Related literature

For the pharmacological and medicinal properties of pyrazole derivatives, see: Baraldi *et al.* (1998 ▶); Bruno *et al.* (1990 ▶); Cottineau *et al.* (2002 ▶); Londershausen (1996 ▶); Chen & Li (1998 ▶); Mishra *et al.* (1998 ▶); Smith *et al.* (2001 ▶). For *sp* ^2^ hybridization, see: Beddoes *et al.*, 1986 ▶). For bond-length data, see: Jin *et al.* (2004 ▶).
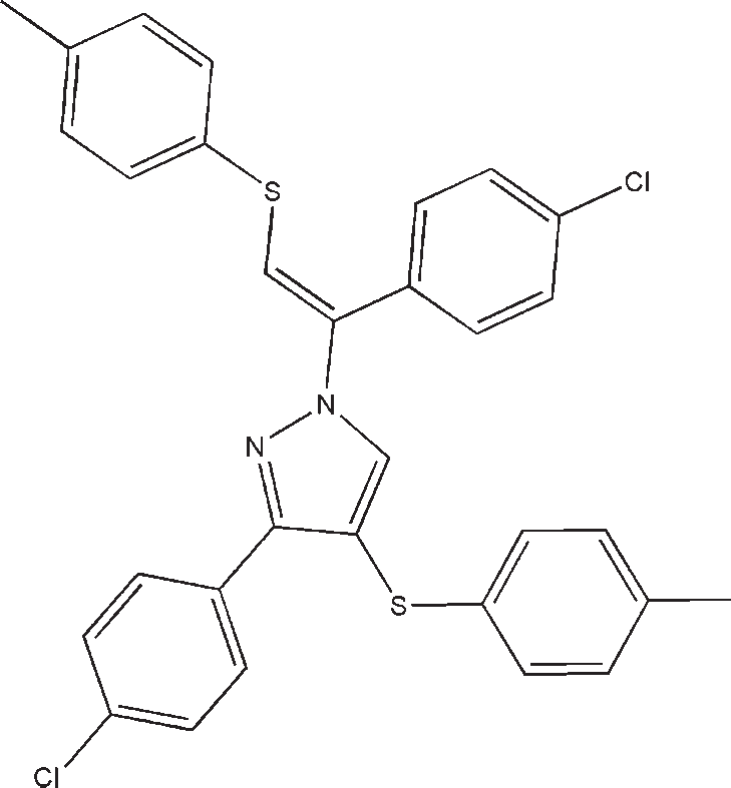


## Experimental

### 

#### Crystal data

C_31_H_24_Cl_2_N_2_S_2_
*M* *_r_* = 559.54
Monoclinic, 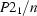

*a* = 9.7515 (2) Å
*b* = 10.2097 (3) Å
*c* = 27.6705 (6) Å
β = 96.402 (1)°
*V* = 2737.69 (11) Å^3^
*Z* = 4
Mo *K*α radiation
μ = 0.41 mm^−1^
*T* = 293 K
0.30 × 0.25 × 0.20 mm

#### Data collection

Bruker Kappa APEXII diffractometer
Absorption correction: multi-scan (*SADABS*; Sheldrick, 2001 ▶) *T*_min_ = 0.883, *T*_max_ = 0.921
38656 measured reflections
9852 independent reflections
5773 reflections with *I* > 2σ(*I*)
*R*_int_ = 0.032

#### Refinement

*R*[*F* ^2^ > 2σ(*F* ^2^)] = 0.052
*wR*(*F* ^2^) = 0.160
*S* = 1.03
9852 reflections
336 parameters
H-atom parameters constrained
Δρ_max_ = 0.54 e Å^−3^
Δρ_min_ = −0.33 e Å^−3^

### 

Data collection: *APEX2* (Bruker, 2004 ▶); cell refinement: *SAINT* (Bruker, 2004 ▶); data reduction: *SAINT*; program(s) used to solve structure: *SHELXS97* (Sheldrick, 2008 ▶); program(s) used to refine structure: *SHELXL97* (Sheldrick, 2008 ▶); molecular graphics: *ORTEP-3* (Farrugia, (1997 ▶)); software used to prepare material for publication: *SHELXL97* and *PLATON* (Spek, 2009 ▶).

## Supplementary Material

Crystal structure: contains datablocks global, I. DOI: 10.1107/S1600536809043293/bt5084sup1.cif

Structure factors: contains datablocks I. DOI: 10.1107/S1600536809043293/bt5084Isup2.hkl

Additional supplementary materials:  crystallographic information; 3D view; checkCIF report

## Figures and Tables

**Table 1:** C—H⋯π interactions (Å, °)

*D*—H⋯*A*	*D*—H	H⋯*A*	*D*⋯*A*	*D*—H⋯*A*
C33—H33*C*⋯*Cg*3^i^	0.96	2.90	3.851 (3)	171
C9—H9⋯*Cg*5^ii^	0.93	3.03	3.839 (2)	147

Symmetry codes: (i) 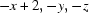; (ii) 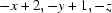. *Cg*3 and *Cg*5 are the centroids of the C14–C19 and C26–C31 rings, respectively.

**Fig. 1.**
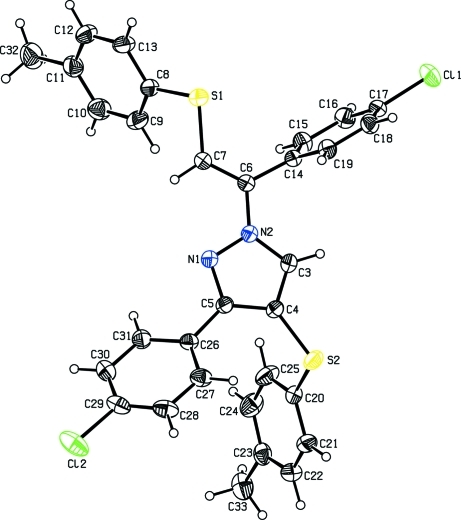
Perspective view of the molecule showing the displacement ellipsoids are drawn at 50% probability level. H atoms have been omitted for clarity.

## supplementary crystallographic information

### Comment

Pyrazole derivatives possess significant antiarrhythmic and sedative (Bruno *et al.*, 1990), hypoglycemic (Cottineau *et al.*, 2002), antiviral (Baraldi *et al.*, 1998), and pesticidal (Londershausen *et al.*, 1996) properties. Some pyrazole derivatives are successfully tested for their antifungal (Chen & Li, 1998), antihistaminic (Mishra *et al.*, 1998) and anti-inflammatory (Smith *et al.*, 2001) activities.

The *ORTEP*  plot of the molecule is shown in Fig. 1. The pyrazole ring adopts a planar conformation. The sum of the bond angles at N2 of the pyrazole ring (360.0°) is in accordance with *sp*2 hybridization (Beddoes *et al.*, 1986). The C—N bond lengths in the pyrazole ring are 1.352 (2) and 1.328 (2) Å, which are shorter than single bond length of 1.443 Å, but longer than a double bond length of 1.269 Å (Jin *et al.*, 2004), indicating electron delocalization. The chlorophenyl rings are twisted from the pyrazole ring at angles of 75.1 (1)° and 39.5 (1)°, respectively. The crystal packing is stabilized by weak C—H···π type of intermolecular interactions in addition to van der Waals forces.

### Experimental

To a mixture of 1-(4-Chlorophenyl) -2-[(4-methylphenyl)sulfanyl]-1-ethanone *N*-(*Z*)-1-(4-chlorophenyl) -2-[(4-methylphenyl)sulfanyl]ethylidenehydrazone (0.003 mole) and 3 ml of dimethyl formamide kept in ice bath at 0°C, phosphorous oxycholride (0.024 mole) was added dropwise for 5–10 minutes. The reaction mixture was then irradiated under microwaves for 30 sec. The process of the reaction was monitored by TLC. After completion of the reaction, the reaction mixture was poured into crushed ice and extracted with dichloromethane. The organic layer was dried over anhydrous sodium sulfate. The different compounds present in the mixture were separated by column chromatography using petroleum ether and ethyl acetate mixture as eluent. This isolated compound was recrystallized in dichloromethane to obtain 3-(4-Chlorophenyl)-1-(*E*)-1-(4-chlorophenyl)-2-[(4-methylphenyl) sulfanyl]ethenyl-4-[(4-methylphenyl)sulfanyl]-1*H*-pyrazole.

### Refinement

All H atoms were positioned geometrically (C—H=0.93–0.96 Å) and allowed to ride on their parent atoms, with 1.5*U*_eq_(C) for methyl H and 1.2 *U*_eq_(C) for other H atoms. The methyl groups were allowed to rotate but not to tip.

### Crystal data

**Table:**

C_31_H_24_Cl_2_N_2_S_2_	*F*(000) = 1160
*M**_r_* = 559.54	*D*_x_ = 1.358 Mg m^−^^3^
Monoclinic, *P*2_1_/*n*	Mo *K*α radiation, λ = 0.71073 Å
Hall symbol: -P 2yn	Cell parameters from 2546 reflections
*a* = 9.7515 (2) Å	θ = 1.5–32.5°
*b* = 10.2097 (3) Å	µ = 0.41 mm^−^^1^
*c* = 27.6705 (6) Å	*T* = 293 K
β = 96.402 (1)°	Block, colourless
*V* = 2737.69 (11) Å^3^	0.30 × 0.25 × 0.20 mm
*Z* = 4	

### Data collection

**Table:**

Bruker Kappa APEXII diffractometer	9852 independent reflections
Radiation source: fine-focus sealed tube	5773 reflections with *I* > 2σ(*I*)
graphite	*R*_int_ = 0.032
ω and φ scans	θ_max_ = 32.5°, θ_min_ = 1.5°
Absorption correction: multi-scan (*SADABS*; Sheldrick, 2001)	*h* = −14→14
*T*_min_ = 0.883, *T*_max_ = 0.921	*k* = −15→15
38656 measured reflections	*l* = −41→39

### Refinement

**Table:**

Refinement on *F*^2^	Primary atom site location: structure-invariant direct methods
Least-squares matrix: full	Secondary atom site location: difference Fourier map
*R*[*F*^2^ > 2σ(*F*^2^)] = 0.052	Hydrogen site location: inferred from neighbouring sites
*wR*(*F*^2^) = 0.160	H-atom parameters constrained
*S* = 1.03	*w* = 1/[σ^2^(*F*_o_^2^) + (0.0723*P*)^2^ + 0.5516*P*] where *P* = (*F*_o_^2^ + 2*F*_c_^2^)/3
9852 reflections	(Δ/σ)_max_ = 0.003
336 parameters	Δρ_max_ = 0.54 e Å^−^^3^
0 restraints	Δρ_min_ = −0.33 e Å^−^^3^

### Special details

**Table:**

Geometry. All e.s.d.'s (except the e.s.d. in the dihedral angle between two l.s. planes) are estimated using the full covariance matrix. The cell e.s.d.'s are taken into account individually in the estimation of e.s.d.'s in distances, angles and torsion angles; correlations between e.s.d.'s in cell parameters are only used when they are defined by crystal symmetry. An approximate (isotropic) treatment of cell e.s.d.'s is used for estimating e.s.d.'s involving l.s. planes.
Refinement. Refinement of *F*^2^ against ALL reflections. The weighted *R*-factor *wR* and goodness of fit *S* are based on *F*^2^, conventional *R*-factors *R* are based on *F*, with *F* set to zero for negative *F*^2^. The threshold expression of *F*^2^ > σ(*F*^2^) is used only for calculating *R*-factors(gt) *etc*. and is not relevant to the choice of reflections for refinement. *R*-factors based on *F*^2^ are statistically about twice as large as those based on *F*, and *R*- factors based on ALL data will be even larger.

### Fractional atomic coordinates and isotropic or equivalent isotropic displacement parameters (Å^2^)

**Table:**

	*x*	*y*	*z*	*U*_iso_*/*U*_eq_	
S1	0.08583 (6)	−0.11974 (5)	0.63584 (2)	0.05549 (15)	
S2	0.25311 (5)	0.35982 (5)	0.42760 (2)	0.05549 (15)	
Cl1	−0.13889 (7)	0.45353 (6)	0.68961 (2)	0.07471 (19)	
Cl2	0.48797 (8)	−0.21313 (9)	0.30187 (2)	0.0905 (2)	
N1	0.23543 (16)	0.02119 (14)	0.49711 (5)	0.0415 (3)	
N2	0.18350 (16)	0.10815 (14)	0.52737 (5)	0.0421 (3)	
C3	0.1835 (2)	0.23137 (18)	0.50948 (7)	0.0472 (4)	
H3	0.1531	0.3062	0.5242	0.057*	
C4	0.23608 (19)	0.22595 (18)	0.46595 (7)	0.0446 (4)	
C5	0.26756 (18)	0.09181 (17)	0.45965 (6)	0.0398 (4)	
C6	0.13934 (19)	0.06576 (17)	0.57196 (6)	0.0410 (4)	
C7	0.1621 (2)	−0.05738 (19)	0.58678 (7)	0.0485 (4)	
H7	0.2196	−0.1107	0.5707	0.058*	
C8	0.2206 (2)	−0.21076 (18)	0.66790 (6)	0.0461 (4)	
C9	0.3584 (2)	−0.1814 (2)	0.66695 (8)	0.0583 (5)	
H9	0.3845	−0.1116	0.6484	0.070*	
C10	0.4568 (3)	−0.2562 (2)	0.69371 (8)	0.0653 (6)	
H10	0.5496	−0.2370	0.6924	0.078*	
C11	0.4224 (3)	−0.3585 (2)	0.72236 (7)	0.0606 (5)	
C12	0.2849 (3)	−0.3848 (2)	0.72369 (8)	0.0614 (6)	
H12	0.2592	−0.4524	0.7433	0.074*	
C13	0.1842 (2)	−0.3134 (2)	0.69661 (7)	0.0566 (5)	
H13	0.0917	−0.3340	0.6976	0.068*	
C14	0.07032 (18)	0.16654 (17)	0.59941 (6)	0.0389 (3)	
C15	0.1380 (2)	0.2157 (2)	0.64224 (7)	0.0495 (4)	
H15	0.2271	0.1877	0.6527	0.059*	
C16	0.0749 (2)	0.3054 (2)	0.66948 (7)	0.0523 (5)	
H16	0.1212	0.3388	0.6980	0.063*	
C17	−0.0571 (2)	0.34490 (18)	0.65410 (6)	0.0459 (4)	
C18	−0.1264 (2)	0.29888 (19)	0.61145 (7)	0.0478 (4)	
H18	−0.2155	0.3273	0.6012	0.057*	
C19	−0.06171 (19)	0.20999 (18)	0.58410 (6)	0.0443 (4)	
H19	−0.1074	0.1789	0.5551	0.053*	
C20	0.4342 (2)	0.38614 (17)	0.43419 (7)	0.0441 (4)	
C21	0.4884 (2)	0.4601 (2)	0.39927 (7)	0.0521 (5)	
H21	0.4301	0.4967	0.3740	0.063*	
C22	0.6289 (2)	0.4800 (2)	0.40176 (8)	0.0549 (5)	
H22	0.6639	0.5305	0.3780	0.066*	
C23	0.7187 (2)	0.42680 (19)	0.43848 (8)	0.0524 (5)	
C24	0.6620 (3)	0.3574 (2)	0.47388 (9)	0.0679 (6)	
H24	0.7199	0.3227	0.4997	0.081*	
C25	0.5222 (2)	0.3377 (2)	0.47219 (8)	0.0649 (6)	
H25	0.4869	0.2914	0.4970	0.078*	
C26	0.32826 (18)	0.02448 (17)	0.42028 (6)	0.0400 (4)	
C27	0.2903 (2)	0.0553 (2)	0.37175 (6)	0.0510 (5)	
H27	0.2310	0.1252	0.3636	0.061*	
C28	0.3405 (2)	−0.0176 (2)	0.33562 (7)	0.0576 (5)	
H28	0.3147	0.0028	0.3031	0.069*	
C29	0.4282 (2)	−0.1200 (2)	0.34765 (7)	0.0553 (5)	
C30	0.4714 (2)	−0.1501 (2)	0.39539 (8)	0.0567 (5)	
H30	0.5336	−0.2178	0.4032	0.068*	
C31	0.4201 (2)	−0.07719 (19)	0.43141 (7)	0.0490 (4)	
H31	0.4480	−0.0969	0.4638	0.059*	
C32	0.5335 (3)	−0.4378 (3)	0.75125 (10)	0.0887 (9)	
H32A	0.5850	−0.4854	0.7294	0.133*	
H32B	0.4918	−0.4983	0.7718	0.133*	
H32C	0.5942	−0.3802	0.7709	0.133*	
C33	0.8720 (2)	0.4392 (3)	0.43899 (11)	0.0728 (7)	
H33A	0.9079	0.3617	0.4253	0.109*	
H33B	0.9136	0.4497	0.4719	0.109*	
H33C	0.8929	0.5140	0.4201	0.109*	

### Atomic displacement parameters (Å^2^)

**Table:**

	*U*^11^	*U*^22^	*U*^33^	*U*^12^	*U*^13^	*U*^23^
S1	0.0571 (3)	0.0534 (3)	0.0597 (3)	0.0077 (2)	0.0230 (2)	0.0147 (2)
S2	0.0492 (3)	0.0490 (3)	0.0690 (3)	0.0058 (2)	0.0096 (2)	0.0254 (2)
Cl1	0.0911 (5)	0.0722 (4)	0.0659 (3)	0.0193 (3)	0.0308 (3)	−0.0139 (3)
Cl2	0.0853 (5)	0.1220 (6)	0.0684 (4)	−0.0011 (4)	0.0276 (3)	−0.0391 (4)
N1	0.0492 (8)	0.0357 (7)	0.0409 (7)	0.0026 (6)	0.0111 (6)	0.0010 (6)
N2	0.0519 (9)	0.0344 (7)	0.0416 (7)	0.0020 (6)	0.0125 (6)	0.0017 (6)
C3	0.0532 (11)	0.0349 (9)	0.0553 (10)	0.0047 (8)	0.0140 (8)	0.0039 (7)
C4	0.0460 (10)	0.0392 (9)	0.0498 (9)	0.0025 (8)	0.0112 (8)	0.0094 (7)
C5	0.0398 (9)	0.0391 (9)	0.0411 (8)	0.0002 (7)	0.0066 (7)	0.0051 (7)
C6	0.0451 (9)	0.0391 (9)	0.0402 (8)	0.0021 (7)	0.0107 (7)	0.0008 (7)
C7	0.0563 (11)	0.0428 (10)	0.0494 (9)	0.0078 (8)	0.0193 (8)	0.0051 (8)
C8	0.0559 (11)	0.0412 (9)	0.0433 (9)	−0.0014 (8)	0.0143 (8)	0.0009 (7)
C9	0.0607 (13)	0.0519 (12)	0.0640 (12)	−0.0077 (10)	0.0141 (10)	0.0108 (9)
C10	0.0556 (13)	0.0749 (15)	0.0660 (13)	−0.0008 (11)	0.0096 (10)	0.0038 (11)
C11	0.0736 (15)	0.0634 (14)	0.0451 (10)	0.0151 (11)	0.0085 (9)	−0.0005 (9)
C12	0.0828 (16)	0.0545 (12)	0.0484 (10)	0.0020 (11)	0.0147 (10)	0.0124 (9)
C13	0.0614 (13)	0.0586 (12)	0.0522 (11)	−0.0069 (10)	0.0174 (9)	0.0101 (9)
C14	0.0424 (9)	0.0360 (8)	0.0393 (8)	−0.0002 (7)	0.0090 (7)	0.0014 (6)
C15	0.0450 (10)	0.0531 (11)	0.0491 (10)	0.0040 (8)	−0.0004 (8)	−0.0048 (8)
C16	0.0611 (12)	0.0535 (11)	0.0415 (9)	−0.0004 (9)	0.0014 (8)	−0.0081 (8)
C17	0.0562 (11)	0.0402 (9)	0.0447 (9)	0.0030 (8)	0.0198 (8)	−0.0002 (7)
C18	0.0411 (10)	0.0457 (10)	0.0574 (10)	0.0025 (8)	0.0090 (8)	−0.0002 (8)
C19	0.0433 (10)	0.0427 (9)	0.0465 (9)	−0.0016 (8)	0.0029 (7)	−0.0048 (7)
C20	0.0502 (10)	0.0361 (9)	0.0469 (9)	0.0048 (7)	0.0091 (8)	0.0047 (7)
C21	0.0570 (12)	0.0542 (11)	0.0459 (9)	0.0034 (9)	0.0086 (8)	0.0114 (8)
C22	0.0569 (12)	0.0552 (12)	0.0549 (11)	−0.0034 (10)	0.0162 (9)	0.0050 (9)
C23	0.0513 (11)	0.0397 (10)	0.0659 (12)	−0.0011 (8)	0.0060 (9)	−0.0104 (8)
C24	0.0593 (13)	0.0685 (15)	0.0720 (14)	0.0010 (11)	−0.0097 (11)	0.0181 (12)
C25	0.0635 (14)	0.0664 (14)	0.0637 (13)	−0.0024 (11)	0.0024 (10)	0.0300 (11)
C26	0.0375 (9)	0.0437 (9)	0.0396 (8)	−0.0027 (7)	0.0075 (6)	0.0031 (7)
C27	0.0421 (10)	0.0679 (13)	0.0428 (9)	0.0010 (9)	0.0043 (7)	0.0072 (8)
C28	0.0469 (11)	0.0878 (16)	0.0382 (9)	−0.0089 (11)	0.0059 (8)	0.0003 (9)
C29	0.0462 (11)	0.0725 (14)	0.0498 (10)	−0.0123 (10)	0.0173 (8)	−0.0154 (9)
C30	0.0568 (12)	0.0572 (12)	0.0578 (11)	0.0074 (10)	0.0141 (9)	−0.0059 (9)
C31	0.0563 (11)	0.0492 (10)	0.0416 (9)	0.0053 (9)	0.0060 (8)	0.0022 (7)
C32	0.100 (2)	0.100 (2)	0.0659 (15)	0.0409 (18)	0.0087 (14)	0.0101 (14)
C33	0.0553 (13)	0.0606 (14)	0.1015 (19)	−0.0034 (11)	0.0041 (12)	−0.0168 (13)

### Geometric parameters (Å, °)

**Table:**

S1—C7	1.7398 (19)	C16—H16	0.9300
S1—C8	1.766 (2)	C17—C18	1.376 (3)
S2—C4	1.7496 (18)	C18—C19	1.378 (3)
S2—C20	1.776 (2)	C18—H18	0.9300
Cl1—C17	1.7340 (18)	C19—H19	0.9300
Cl2—C29	1.736 (2)	C20—C25	1.373 (3)
N1—C5	1.328 (2)	C20—C21	1.377 (3)
N1—N2	1.3565 (19)	C21—C22	1.379 (3)
N2—C3	1.352 (2)	C21—H21	0.9300
N2—C6	1.419 (2)	C22—C23	1.376 (3)
C3—C4	1.362 (3)	C22—H22	0.9300
C3—H3	0.9300	C23—C24	1.374 (3)
C4—C5	1.418 (3)	C23—C33	1.500 (3)
C5—C26	1.467 (2)	C24—C25	1.373 (3)
C6—C7	1.333 (3)	C24—H24	0.9300
C6—C14	1.484 (2)	C25—H25	0.9300
C7—H7	0.9300	C26—C31	1.383 (3)
C8—C9	1.380 (3)	C26—C27	1.389 (2)
C8—C13	1.385 (3)	C27—C28	1.379 (3)
C9—C10	1.377 (3)	C27—H27	0.9300
C9—H9	0.9300	C28—C29	1.369 (3)
C10—C11	1.376 (3)	C28—H28	0.9300
C10—H10	0.9300	C29—C30	1.375 (3)
C11—C12	1.372 (4)	C30—C31	1.381 (3)
C11—C32	1.508 (3)	C30—H30	0.9300
C12—C13	1.376 (3)	C31—H31	0.9300
C12—H12	0.9300	C32—H32A	0.9600
C13—H13	0.9300	C32—H32B	0.9600
C14—C19	1.383 (3)	C32—H32C	0.9600
C14—C15	1.385 (2)	C33—H33A	0.9600
C15—C16	1.374 (3)	C33—H33B	0.9600
C15—H15	0.9300	C33—H33C	0.9600
C16—C17	1.371 (3)		
			
C7—S1—C8	103.04 (9)	C19—C18—H18	120.5
C4—S2—C20	102.51 (9)	C18—C19—C14	120.68 (17)
C5—N1—N2	105.26 (13)	C18—C19—H19	119.7
C3—N2—N1	111.58 (14)	C14—C19—H19	119.7
C3—N2—C6	127.92 (15)	C25—C20—C21	118.63 (19)
N1—N2—C6	120.49 (14)	C25—C20—S2	123.61 (16)
N2—C3—C4	107.54 (16)	C21—C20—S2	117.76 (14)
N2—C3—H3	126.2	C20—C21—C22	120.14 (18)
C4—C3—H3	126.2	C20—C21—H21	119.9
C3—C4—C5	104.82 (15)	C22—C21—H21	119.9
C3—C4—S2	125.11 (15)	C23—C22—C21	121.67 (19)
C5—C4—S2	130.04 (14)	C23—C22—H22	119.2
N1—C5—C4	110.80 (15)	C21—C22—H22	119.2
N1—C5—C26	118.34 (15)	C24—C23—C22	117.2 (2)
C4—C5—C26	130.85 (15)	C24—C23—C33	121.1 (2)
C7—C6—N2	119.92 (16)	C22—C23—C33	121.7 (2)
C7—C6—C14	124.46 (16)	C25—C24—C23	121.9 (2)
N2—C6—C14	115.62 (14)	C25—C24—H24	119.1
C6—C7—S1	120.93 (15)	C23—C24—H24	119.1
C6—C7—H7	119.5	C20—C25—C24	120.4 (2)
S1—C7—H7	119.5	C20—C25—H25	119.8
C9—C8—C13	119.17 (19)	C24—C25—H25	119.8
C9—C8—S1	123.22 (15)	C31—C26—C27	118.79 (17)
C13—C8—S1	117.56 (16)	C31—C26—C5	119.41 (15)
C10—C9—C8	119.4 (2)	C27—C26—C5	121.71 (17)
C10—C9—H9	120.3	C28—C27—C26	120.04 (19)
C8—C9—H9	120.3	C28—C27—H27	120.0
C11—C10—C9	122.1 (2)	C26—C27—H27	120.0
C11—C10—H10	119.0	C29—C28—C27	119.92 (18)
C9—C10—H10	119.0	C29—C28—H28	120.0
C12—C11—C10	117.8 (2)	C27—C28—H28	120.0
C12—C11—C32	121.8 (2)	C28—C29—C30	121.33 (19)
C10—C11—C32	120.4 (2)	C28—C29—Cl2	119.50 (16)
C11—C12—C13	121.5 (2)	C30—C29—Cl2	119.16 (18)
C11—C12—H12	119.3	C29—C30—C31	118.5 (2)
C13—C12—H12	119.3	C29—C30—H30	120.8
C12—C13—C8	120.0 (2)	C31—C30—H30	120.8
C12—C13—H13	120.0	C30—C31—C26	121.40 (18)
C8—C13—H13	120.0	C30—C31—H31	119.3
C19—C14—C15	118.97 (16)	C26—C31—H31	119.3
C19—C14—C6	121.71 (15)	C11—C32—H32A	109.5
C15—C14—C6	119.30 (16)	C11—C32—H32B	109.5
C16—C15—C14	120.76 (18)	H32A—C32—H32B	109.5
C16—C15—H15	119.6	C11—C32—H32C	109.5
C14—C15—H15	119.6	H32A—C32—H32C	109.5
C17—C16—C15	119.21 (17)	H32B—C32—H32C	109.5
C17—C16—H16	120.4	C23—C33—H33A	109.5
C15—C16—H16	120.4	C23—C33—H33B	109.5
C16—C17—C18	121.34 (17)	H33A—C33—H33B	109.5
C16—C17—Cl1	119.20 (15)	C23—C33—H33C	109.5
C18—C17—Cl1	119.46 (16)	H33A—C33—H33C	109.5
C17—C18—C19	119.02 (18)	H33B—C33—H33C	109.5
C17—C18—H18	120.5		
			
C5—N1—N2—C3	−0.3 (2)	C19—C14—C15—C16	0.6 (3)
C5—N1—N2—C6	−179.36 (16)	C6—C14—C15—C16	−177.49 (18)
N1—N2—C3—C4	0.4 (2)	C14—C15—C16—C17	0.8 (3)
C6—N2—C3—C4	179.37 (18)	C15—C16—C17—C18	−1.5 (3)
N2—C3—C4—C5	−0.3 (2)	C15—C16—C17—Cl1	177.69 (16)
N2—C3—C4—S2	178.16 (14)	C16—C17—C18—C19	0.8 (3)
C20—S2—C4—C3	108.01 (18)	Cl1—C17—C18—C19	−178.36 (15)
C20—S2—C4—C5	−73.92 (19)	C17—C18—C19—C14	0.6 (3)
N2—N1—C5—C4	0.10 (19)	C15—C14—C19—C18	−1.3 (3)
N2—N1—C5—C26	179.47 (15)	C6—C14—C19—C18	176.76 (17)
C3—C4—C5—N1	0.1 (2)	C4—S2—C20—C25	−18.3 (2)
S2—C4—C5—N1	−178.23 (15)	C4—S2—C20—C21	162.26 (16)
C3—C4—C5—C26	−179.13 (18)	C25—C20—C21—C22	2.6 (3)
S2—C4—C5—C26	2.5 (3)	S2—C20—C21—C22	−177.86 (16)
C3—N2—C6—C7	−171.8 (2)	C20—C21—C22—C23	0.4 (3)
N1—N2—C6—C7	7.1 (3)	C21—C22—C23—C24	−2.7 (3)
C3—N2—C6—C14	7.6 (3)	C21—C22—C23—C33	174.9 (2)
N1—N2—C6—C14	−173.51 (15)	C22—C23—C24—C25	2.0 (4)
N2—C6—C7—S1	−169.62 (14)	C33—C23—C24—C25	−175.6 (2)
C14—C6—C7—S1	11.0 (3)	C21—C20—C25—C24	−3.2 (4)
C8—S1—C7—C6	−138.30 (17)	S2—C20—C25—C24	177.26 (19)
C7—S1—C8—C9	27.9 (2)	C23—C24—C25—C20	0.9 (4)
C7—S1—C8—C13	−154.47 (16)	N1—C5—C26—C31	−37.2 (2)
C13—C8—C9—C10	1.3 (3)	C4—C5—C26—C31	142.1 (2)
S1—C8—C9—C10	178.90 (17)	N1—C5—C26—C27	139.28 (18)
C8—C9—C10—C11	−1.3 (4)	C4—C5—C26—C27	−41.5 (3)
C9—C10—C11—C12	0.0 (3)	C31—C26—C27—C28	2.1 (3)
C9—C10—C11—C32	−179.8 (2)	C5—C26—C27—C28	−174.36 (18)
C10—C11—C12—C13	1.3 (3)	C26—C27—C28—C29	−0.3 (3)
C32—C11—C12—C13	−178.9 (2)	C27—C28—C29—C30	−2.0 (3)
C11—C12—C13—C8	−1.3 (3)	C27—C28—C29—Cl2	178.72 (16)
C9—C8—C13—C12	−0.1 (3)	C28—C29—C30—C31	2.3 (3)
S1—C8—C13—C12	−177.79 (17)	Cl2—C29—C30—C31	−178.35 (16)
C7—C6—C14—C19	−108.0 (2)	C29—C30—C31—C26	−0.5 (3)
N2—C6—C14—C19	72.6 (2)	C27—C26—C31—C30	−1.7 (3)
C7—C6—C14—C15	70.0 (3)	C5—C26—C31—C30	174.82 (18)
N2—C6—C14—C15	−109.32 (19)		

### Hydrogen-bond geometry (Å, °)

**Table:**

*D*—H···*A*	*D*—H	H···*A*	*D*···*A*	*D*—H···*A*
C33—H33C···Cg3^i^	0.96	2.90	3.851 (3)	171
C9—H9···Cg5^ii^	0.93	3.03	3.839 (2)	147

Symmetry codes: (i) −*x*+2, −*y*, −*z*; (ii) −*x*+2, −*y*+1, −*z*.

## References

[bb1] Baraldi, P. G., Manfredini, S., Romagnoli, R., Stevanato, L., Zaid, A. N. & Manservigi, R. (1998). *Nucleosides Nucleotides*, **17**, 2165–2171.

[bb2] Beddoes, R. L., Dalton, L., Joule, T. A., Mills, O. S., Street, J. D. & Watt, C. I. F. (1986). *J. Chem. Soc. Perkin Trans. 2*, pp. 787–797.

[bb3] Bruker (2004). *APEX2* and *SAINT* Bruker AXS Inc., Madison, Wisconsin, USA.

[bb4] Bruno, O., Bondavalli, F., Ranise, A., Schenone, P., Losasso, C., Cilenti, L., Matera, C. & Marmo, E. (1990). *Farmaco*, **45**, 147–66. 2133992

[bb5] Chen, H. S. & Li, Z. M. (1998). *Chem. J. Chin. Univ.* **19**, 572–576.

[bb6] Cottineau, B., Toto, P., Marot, C., Pipaud, A. & Chenault, J. (2002). *Bioorg. Med. Chem.* **12**, 2105–2108. 10.1016/s0960-894x(02)00380-3 12127514

[bb7] Farrugia, L. J. (1997). *J. Appl. Cryst.* **30**, 565.

[bb8] Jin, Z.-M., Li, L., Li, M.-C., Hu, M.-L. & Shen, L. (2004). *Acta Cryst.* C**60**, o642–o643. 10.1107/S0108270104016130 15345843

[bb9] Londershausen, M. (1996). *Pestic. Sci.* **48**, 269–274.

[bb10] Mishra, P. D., Wahidullah, S. & Kamat, S. Y. (1998). *Indian J. Chem. Sect. B*, **37**, 199–200.

[bb11] Sheldrick, G. M. (2001). *SADABS* University of Göttingen, Germany.

[bb12] Sheldrick, G. M. (2008). *Acta Cryst.* A**64**, 112–122. 10.1107/S0108767307043930 18156677

[bb13] Smith, S. R., Denhardt, G. & Terminelli, C. (2001). *Eur. J. Pharmacol.* **432**, 107–119. 10.1016/s0014-2999(01)01477-7 11734194

[bb14] Spek, A. L. (2009). *Acta Cryst.* D**65**, 148–155. 10.1107/S090744490804362X PMC2631630 19171970

